# Biological significance of FoxN1 gain-of-function mutations during T and B lymphopoiesis in juvenile mice

**DOI:** 10.1038/cddis.2014.432

**Published:** 2014-10-09

**Authors:** L Ruan, Z Zhang, L Mu, P Burnley, L Wang, B Coder, Q Zhuge, D-M Su

**Affiliations:** 1 Zhejiang Provincial Key Laboratory of Aging and Neurological Disorder Research, First Affiliated Hospital, Wenzhou Medical University, Wenzhou, China; 2 Department of Cell Biology and Immunology, University of North Texas Health Science Center at Fort Worth, Fort Worth, TX, USA

## Abstract

*FoxN1* is cell-autonomously expressed in skin and thymic epithelial cells (TECs), essential for their development. Inborn mutation of *FoxN1* results in hair follicle and TEC development failure, whereas insufficient postnatal FoxN1 expression induces thymic atrophy, resulting in declined T lymphopoiesis. Although upregulating FoxN1 expression in the aged FoxN1-declined thymus rejuvenates T lymphopoiesis, whether its over- and ectopic-expression in early life is beneficial for T lymphopoiesis is unknown. Using our newly generated *Rosa26*-*STOP*^flox^–*FoxN1* mice, in which over- and ectopic-expression of FoxN1 can be induced by various promoter-driven Cre-mediated deletions of the roadblock *STOP*^flox^ in early life, we found that K14Cre-mediated inborn FoxN1 overexpression induced neonatal lethality, exhibited abnormal permeability in the skin and abnormal nursing. Ubiquitous deletion of the *STOP*^flox^ mediated by progressive uCreER^T^ leakage in juvenile mice affected thymus and bone marrow normality, resulting in an increased ratio of medullary/cortical TECs, along with declined T and B lymphopoiesis. Although the K5CreER^T^-mediated FoxN1 overexpression mice had a normal lifespan, induction of K5CreER^T^ activation in juveniles adversely influenced total thymoycte development and produced ichthyosis-like skin. Therefore, FoxN1 has temporal and tissue-specific activity. Over- and ectopic-expression of FoxN1 in early life adversely influence immature TEC, T and B cell, and skin epithelial development.

Transcription factor *FoxN1* is an epithelial cell-autonomous gene, predominantly regulating development of thymic epithelial cells (TECs) and skin keratinocytes.^1^ Inborn *FoxN1* mutation results in thymic and hair follicle epithelial development failure^2, 3, 4^ associated with primary immune deficiency^5, 6, 7^ and hairless nude skin.^8, 9, 10^ Insufficient FoxN1 expression in postnatal life results in accelerated thymic aging.^11, 12, 13^ Although elevating FoxN1 expression in the aged thymus can reinstate TEC homeostasis and induce thymic functional rejuvenation,^12,14,15^ whether FoxN1 activity is sensitive to genetic dosage, tissue, and temporal-specificity is still unclear. Mounting evidence shows that dosage of FoxN1 is indeed important for postnatal thymic homeostasis. When dosage of FoxN1 is slightly elevated or depressed, the status of the postnatal thymus is significantly influenced. It is known that the thymus in wild-type (WT) young mice with a full dosage of FoxN1 is completely normal, whereas it is completely abnormal in *FoxN1*-null (inborn mutation, termed ‘*nu/nu*') nude mice. In between these two extremes, the *FoxN1*-null heterozygote (*nu*/+) mouse has a 50% reduced genetic dosage compared to its WT littermates. Although the 50% dosage of FoxN1 is sufficient to induce TEC patterning^4^ during thymic organogenesis, the *nu/+* mice have certain defects in thymic size and thymocyte number,^16,17^ particularly as age increases, compared with WT littermates. When FoxN1 was reduced by ~30% compared to its WT levels in the postnatal thymus, thymic atrophy was significantly induced.^11,18^ On the other hand, when FoxN1 dosage was increased by infusing minuscule amounts of vector-carried *FoxN1* cDNA into a naturally aged thymus, it resulted in significant thymic rejuvenation.^12^ Using a Keratin(K)14 promoter-driven FoxN1 transgenic (Tg) mouse model the overexpression of FoxN1-attenuated age-associated thymic involution.^14^ Upregulating FoxN1 specifically in TECs (via inducible *FoxN1ER* Tg) in the aged fully involuted thymus confirmed the effect of reversing the involution.^15^ These experiments demonstrated that age-related thymic involution is causally associated with the loss of FoxN1.^12,19^ However, whether the dosage of FoxN1 can be regarded as ‘the more, the better' for promoting thymic development is arguable. In addition, another recent report using the same K14-FoxN1 transgenic mice showed that FoxN1 expressed in the bone marrow (BM) of K14^+^ stroma, which is not a prominent site of FoxN1 expression, promoted T-lineage production, but inhibited B-lineage production.^20^ Therefore, another question about the role of FoxN1 in nonprominent locations arises.

We addressed these questions using an inducible FoxN1 expression mouse model—the *Rosa26-STOP*^flox^–*FoxN1* transgenic mouse.^1^ In this mouse, the *FoxN1* cDNA is driven by the ubiquitously expressed *Rosa26* promoter, and its expression is enhanced by a composite of CMV immediate-early gene enhancer/chicken *β*-actin promoter (pCAG).^21, 22, 23^ This induces overexpression of exogenous *FoxN1* after Cre-mediated deletion of the roadblock *STOP*^flox^. We found that FoxN1 over- and ectopic-expression (or nonprominent location expression) in early life stages adversely affected the development of the skin, thymus and T cells, as well as B cells, whereas K5CreER^T^-mediated FoxN1 overexpression in adult mice did not cause any observable defects, and can probably be expected to attenuate age-related thymic involution as reported.^14,15^ Therefore, FoxN1 exhibits both temporal and tissue-specific activity.

## Results

### The characteristics of *Rosa26-STOP*^flox^–*FoxN1* mouse model

We generated an inducible exogenous FoxN1 expression mouse model, the *Rosa26*-*STOP*^flox^–*FoxN1* transgenic mouse, to study the biological significance of over- and ectopic-expression of FoxN1.^1^ In this mouse, the flag-*FoxN1* cDNA (kindly provided by Dr. Brissette)^24^ driven by a pCAG promoter^21, 22, 23^ (kindly provided by Dr. McMahon) was inserted into the *Rosa26* locus. This fragment was book-ended by a *STOP*^flox^ roadblock cassette and IRES-GFP reporter gene, respectively (Supplementary Figure S1A). The DNA construct was verified by sequencing. This makes conditional expression of the *FoxN1* transgene controlled by tissue-specific Cre/CreER^T^ genes. We first selected K14Cre to mediate the removal of the roadblock, because K14^+^ epithelial cells are epithelial progenitor cells in the skin, lung, and breast epithelial basal layer, and FoxN1 is a regulator that controls skin and thymic epithelial progenitor cell differentiation. We found that newborn mice with both *Rosa26*-*STOP*^flox^–*FoxN1* and K14Cre transgenes (termed *FoxN1*^Tg^K14Cre^+^) had strong FoxN1 and GFP expression (Supplementary Figures S1B and C) in the skin and thymus.^1^ To verify the targeted locus, Southern blot for *Rosa26*-*STOP*^flox^–*FoxN1* mouse genomic DNA was performed. The results of correct genomic DNA size (with EcoRV digestion) are shown in Supplementary Figure S1D. To verify whether this exogenous *FoxN1* is functional, we crossbred the *Rosa26*-*STOP*^flox^–*FoxN1* mice with *FoxN1*^flox^-K5CreER^T^ conditional gene knockout (cKO) mice^11^ to get *FoxN1*^Tg^*-FoxN1*^flox^ cKO mice. In this mouse, tamoxifen (TM) induction via K5CreER^T^^25^ conditionally knocks out endogenous *FoxN1* while inducing exogenous *FoxN1* expression. We found that the *FoxN1*^Tg^-*FoxN1*^flox^ cKO mice had a normal thymus (data not shown), whereas *FoxN1*^flox^ cKO mice alone had an atrophied thymus,^11^ indicating *FoxN1*^Tg^ has normal FoxN1 activity that can compensate for the loss of *FoxN1*^flox^ in the thymus.

Unfortunately, *FoxN1*^Tg^K14Cre^+^ mice cannot survive for more than 24 h after birth. The mice had opened eyes from later gestation until birth compared to their *FoxN1*^Tg^K14Cre^−^ (nonexogenous *FoxN1* expression) littermates (Supplementary Figure S1E).

### Neonatal *FoxN1*^Tg^K14Cre^+^ mice had increased skin permeability and defect in nursing

**Figure 1:**
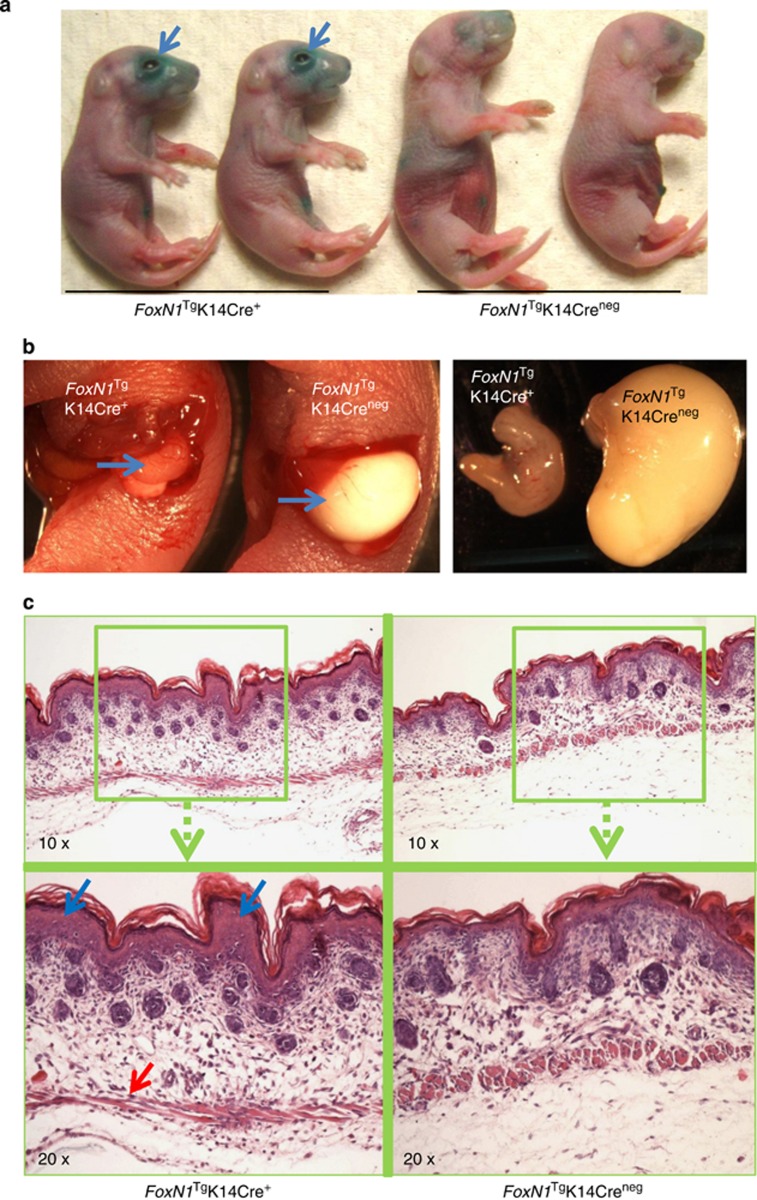
FoxN1 overexpression mediated by K14Cre^+^-induced defects in skin and nursing in newborn mice. (**a**) Skin permeability assay with X-gal shows increased permeability, mostly focused around the eyes (blue arrows) of *FoxN1*^Tg^K14Cre^+^ newborn mice; (**b**) stomachs (blue arrows) of *FoxN1*^Tg^K14Cre^+^ (left) and *FoxN1*^Tg^K14Cre^−^ (right) newborn mice, with little to no milk in the stomach of *FoxN1*^Tg^K14Cre^+^ newborn mouse; and (**c**) skin histological assay (H&E staining) shows increased thickness in the epidermal layer (blue arrow) and flattening in the muscle layer (red arrow) of *FoxN1*^Tg^K14Cre^+^ newborn mice

In order to determine what causes *FoxN1*^Tg^K14Cre^+^ neonatal death, we focused on water intake and retention, because our neonatal mice share similar phenotypes with the involucrin promoter-driven *FoxN1* transgenic neonatal mice,^24^ which possess open eyes at birth and neonatal lethal phenotype with dehydration. Using an d-galactopyranoside (X-gal) staining approach, which is one of the skin barrier function assays used for determining skin permeability,^26^ we found that *FoxN1*^Tg^K14Cre^+^ newborn skin permeability was increased, mostly focused around the eye region (arrows in Figure 1a). The increased region of skin permeability was consistent with the open-eye phenotype. We also found that these newborn mice cannot nurse by checking their stomach (Figure 1b). In addition, the microstructure of *FoxN1*^Tg^K14Cre^+^ newborn skin showed increased thickness in the epidermal layer (blue arrow in Figure 1c) and flattened structure in the muscle layer (red arrow in Figure 1c), which are related to dehydration and flexibility in the stratum corneum.^27^ The structure looks similar to the changes seen in congenital ichthyosis,^27^ but not as severe. These phenotypes suggest that FoxN1 overexpression in K14^+^ epithelium of the skin induces developmental mutations that result in an increase in skin permeability and defect in nursing. Therefore, *FoxN1*^Tg^K14Cre^+^ neonates die from dehydration.

### Overexpression of exogenous *FoxN1*Tg, mediated by uCreER^T^ in juveniles, adversely affects thymus juvenile stage maturation

We next asked whether FoxN1 overexpression influences thymus development and T lymphopoiesis, as the main role of FoxN1 is the regulation of TEC development, thereby ensuring thymus and T cell development. We observed thymic microstructure and thymocyte profile of *FoxN1*^Tg^K14Cre^+^ neonatal mice (Supplementary Figure S2), but did not find any differences compared to their *FoxN1*^Tg^K14Cre^−^ littermate controls. Upon crossbreeding *Rosa26*-*STOP*^flox^–*FoxN1* mice with uCreER^T^ mice,^11,28^ the neonates exhibited the same features as that of the *FoxN1*^Tg^K14Cre^+^ neonates: a normal thymic microstructure and thymocyte profile, but the *FoxN1*^Tg^uCreER^T+^ neonatal mice did not have a lethal phenotype. However, *FoxN1*^Tg^uCreER^T+^ mice cannot survive for more than 3 weeks following birth. Furthermore, thymic and thymocyte phenotypes underwent dramatic changes ~20 days after birth, likely as a result of the progressive expression of exogenous *FoxN1*^Tg^ in the juvenile stage, mediated by a progressive leakage of Cre-recombinase from uCreER^T^ due to incomplete ER blockage *in vivo* with age, which was confirmed in our previous publications^1,12^ and by an other group.^29^

**Figure 2:**
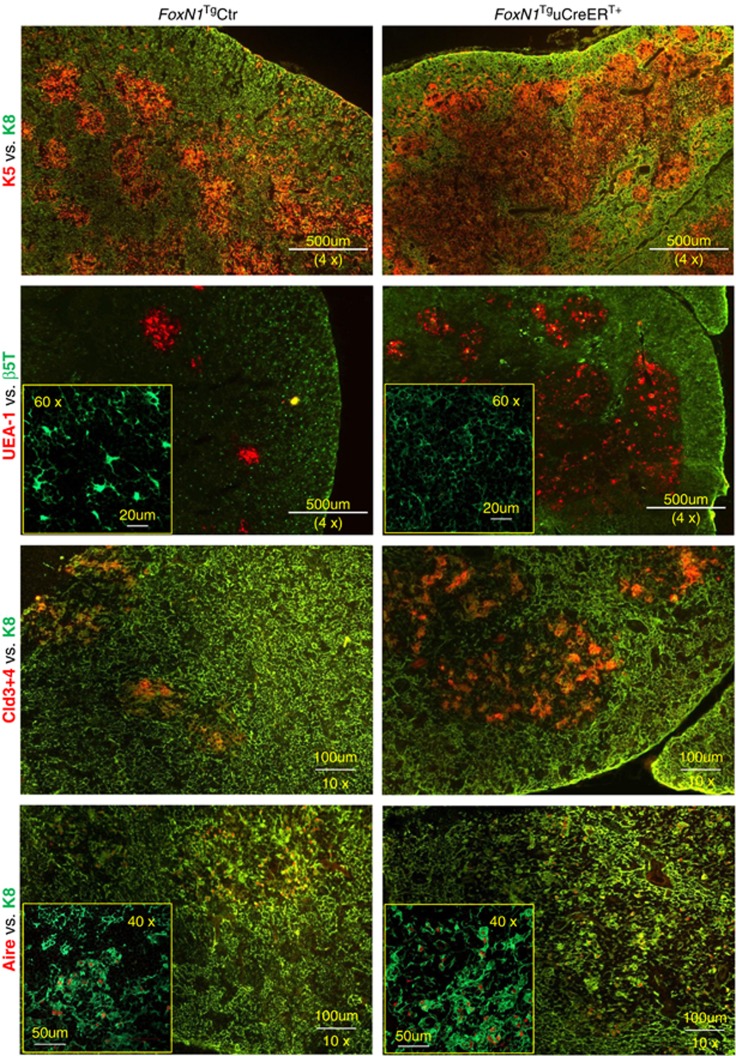
Overexpression of FoxN1 mediated by uCreER^T+^ in juvenile mice induced medullary-biased abnormality of thymic microstructure. Representative immunofluorescence staining shows the thymuses of *FoxN1*^Tg^uCreER^T+^ juvenile (~20 days after birth) mice (right panels) had infused thymic medullary islets (top panels), an increase in UEA-1^+^ and Cld3,4^+^ TECs (panels in middle two rows), an increase in Aire^+^ mTECs (bottom panels), and dimness of 5*β*t^+^ bright dendritic-shaped spots (second row from top), compared to their normal littermate controls (left panels). This experiment was repeated three times with at least three animals in each group producing consistent results

**Figure 3:**
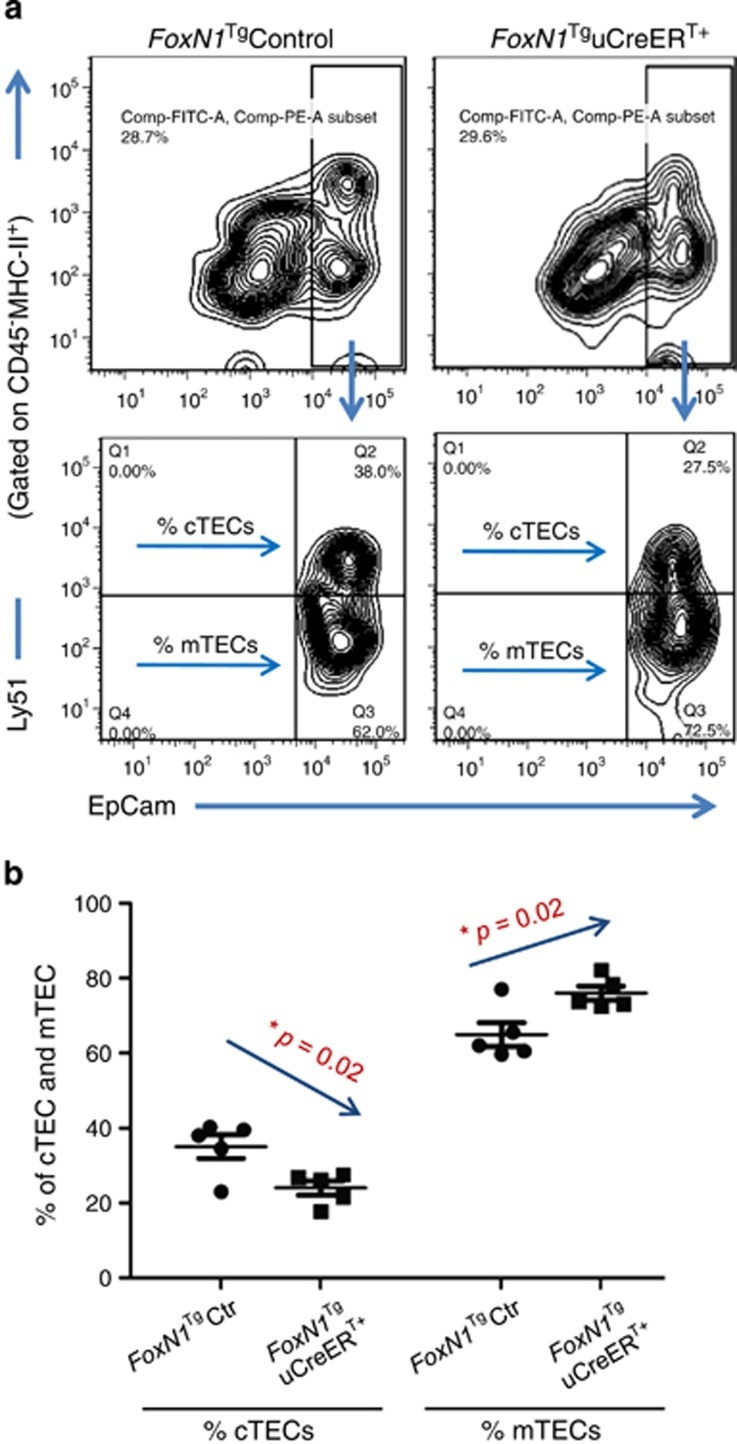
Ratio of mTEC/cTEC with flow cytometry assay confirms medullary-biased development in *FoxN1*^Tg^uCreER^T+^ juvenile mice. (**a**) Contour plots show representative flow cytometric gates of mTEC and cTEC subpopulation; and (**b**) summarized result from five mice in each group shows decreased percentages of cTECs and increased percentages of mTECs in *FoxN1*^Tg^uCreER^T+^ juvenile (~20 days after birth) mice. *=statistically significant

Evidence has shown that the thymus, particularly the thymic medulla, continuously undergoes maturation in the juvenile stage.^30^ We found that ubiquitous FoxN1 overexpression in the juveniles indeed promoted a thymic medulla-skewed maturation, which resulted in an infusion of thymic medullary islets to occupy a large region (Figure 2, top panels), and increased the proportion of claudin-3, -4 (Cld3,4^+^) and UEA-1^+^ TECs (Figure 2 middle rows), which represent immature (Cld3,4)^31^ and mature organized mTECs (UEA-1),^32^ respectively, as well as an increase in Aire^+^ mTECs (Figure 2 bottom panels). However, the trade-off for enhanced mTEC maturation was defective cTECs, as exhibited by diminished *β*5t^+^ dendritic-shaped bright spots that are normally observed in the *FoxN1*^Tg^uCreER^−^ control thymus (Figure 2, left panel in the second row). *β*5t is considered a cTEC biomarker^33^ and is essential for the positive selection of T cell receptor.^34,35^ Flow cytometry analysis of the ratio of mTECs/cTECs further confirmed that the proportion of mTECs were increased at the expense of cTECs (Figure 3).

**Figure 4:**
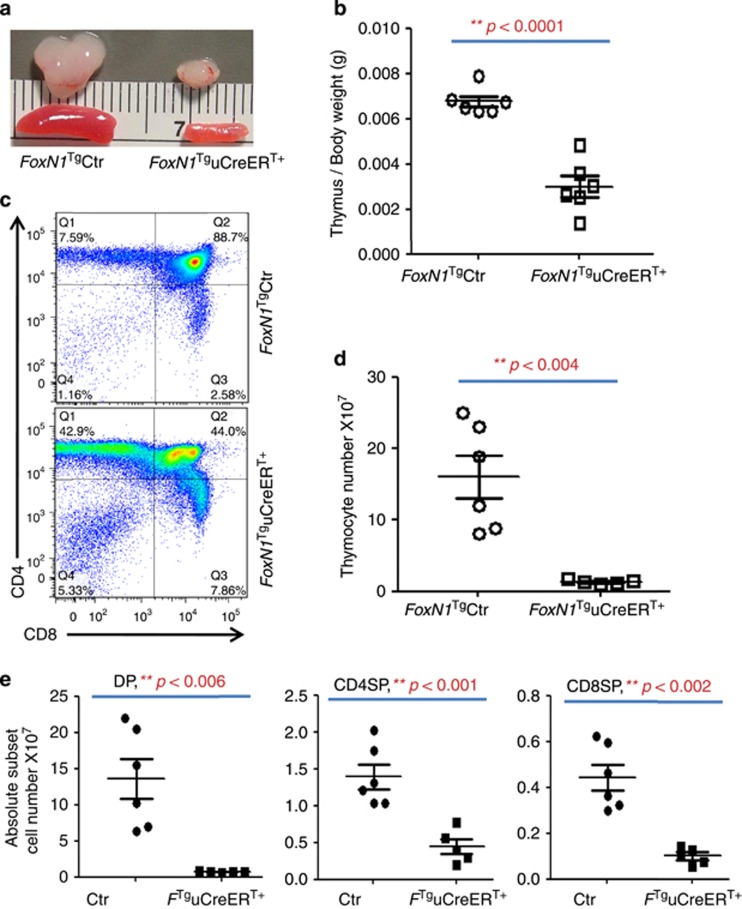
uCreER^T+^-mediated FoxN1 overexpression reduced the thymus and spleen, and cell numbers, altered thymocyte profile. (**a**) A representative image of the thymus and spleen of *FoxN1*^Tg^uCreER^T+^ juvenile (~20 days after birth) mice and their littermate controls; (**b**) the thymus/body weight from seven randomly selected animals in each group; (**c**) a representative CD4 *versus* CD8 profile with flow cytometry assay; (**d**) absolute total thymocyte number; and (**e**) absolute CD4^+^8^+^ DP, CD4SP, and CD8SP thymocyte numbers (each spot represents one animal). **=statistically very significant

On checking the thymocyte profile, we found that the thymic size (Figure 4a) and weight (Figure 4b) were reduced in the FoxN1 overexpression group. Furthermore, the thymocyte profile was obviously changed compared with control group (Figure 4c), with significantly decreased absolute cell number of total thymocytes (Figure 4d) and all subpopulations (Figure 4e), especially CD4^+^8^+^ double positive cells. The results indicate that FoxN1 overexpression in juvenile mice adversely affected thymocyte development by influencing thymic microstructure maturation and potential ectopic expression of FoxN1 in hematopoietic cells via uCreER^T^ mediation.

### *FoxN1*^Tg^uCreER^T+^ juveniles showed deterioration of T cell function in the periphery

**Figure 5:**
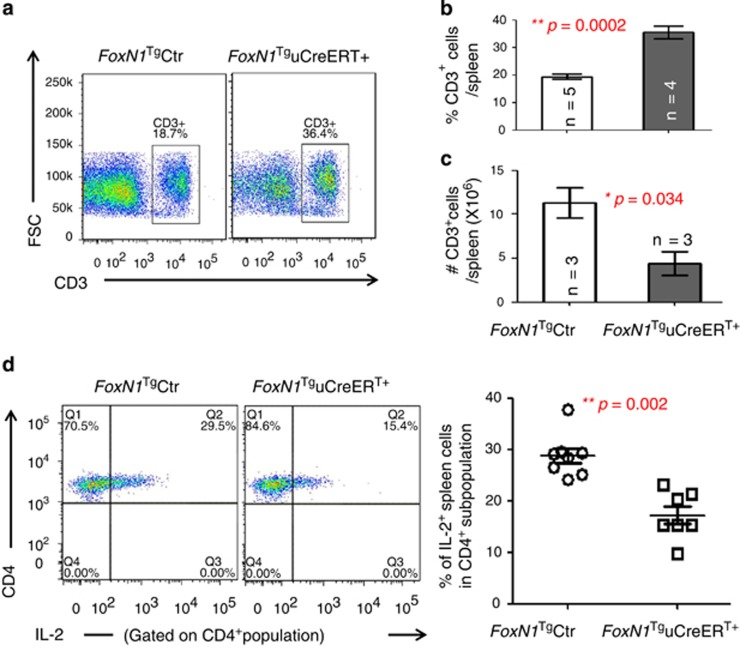
T cells in *FoxN1*^Tg^uCreER^T+^ juvenile mice increased %, reduced absolute number, and declined responsiveness. (**a**) Representative flow cytometric gates of splenic CD3^+^ T cells; (**b**) summarized results of the percentages of CD3^+^ T cells in the spleen; (**c**) summarized results of absolute CD3^+^ T cell number per spleen (*n*=number of animals in panels B and C); and (**d**) dot plots on the left show a representative flow cytometric experiment of splenic IL-2^+^ T cells in CD4^+^ population gate in response to costimulation from anti-CD3*ɛ* and anti-CD28, whereas the panel on the right shows summary of CD4^+^IL-2^+^ T cells in the spleen in response to costimulation from anti-CD3*ɛ* and anti-CD28 (seven animals in each group). *=statistically significant; **=statistically very significant

We asked whether reduced thymocyte number, resulting from the overexpression of exogenous FoxN1, affects mature T cell function in the periphery by testing CD4 splenic T cell responsiveness to anti-CD3*ɛ* and anti-CD28 costimulation. We noticed that the proportion of splenic T cells were significantly increased (Figures 5a and b), whereas the absolute cell number was significantly decreased (Figure 5c). IL-2 production was significantly decreased in CD4^+^ splenic T cells in response to costimulation (Figure 5d), implying that the peripheral T cells were functionally impaired in the *FoxN1*^Tg^uCreER^T+^ juvenile mice.

### Ectopic expression of exogenous *FoxN1*^Tg^, mediated by uCreER^T^ in juvenile BM, adversely affects B cell development

**Figure 6:**
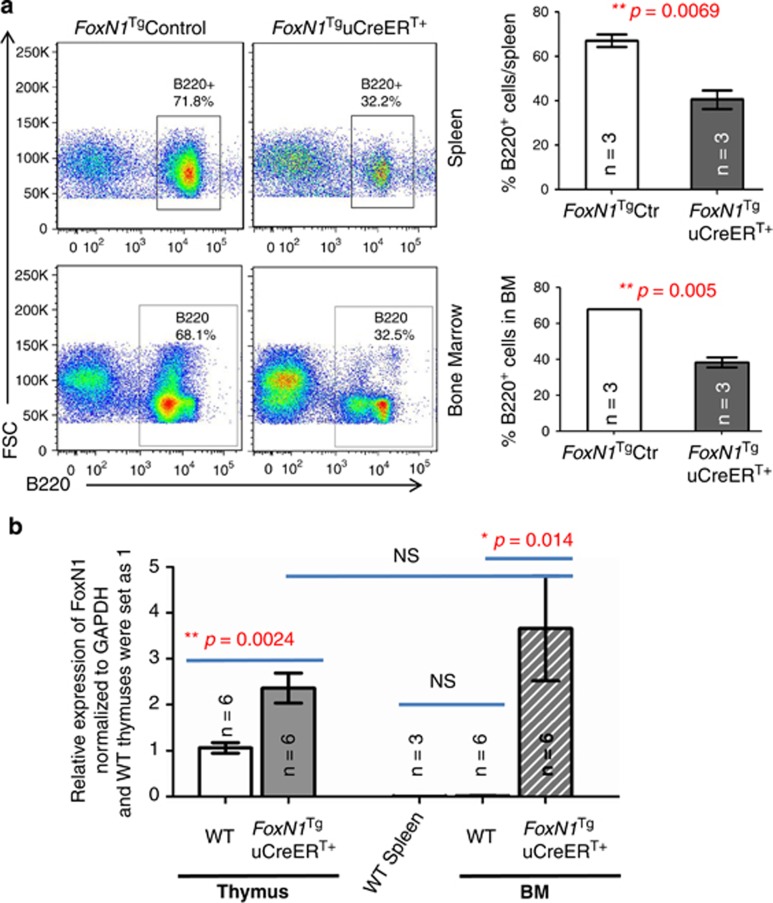
Defective B cell development in the spleen and BM of *FoxN1*^Tg^uCreER^T+^ juvenile mice. (**a**) Flow cytometric dot plots on the left show representative gates on splenic (top panels) and BM (bottom panels) B220^+^ B cells, whereas the panels on the right show summarized results of the percentages of B220^+^ B cells in the spleen (top bar graph) and BM (bottom bar graph; and (**b**) FoxN1 expression in the thymus and BM of *FoxN1*^Tg^uCreER^T+^ juvenile mice tested by real-time RT-PCR assay; (*n*=number of animals). *=statistically significant; **=statistically very significant

We noticed that the *FoxN1*^Tg^uCreER^T+^ juvenile mice had dramatically reduced mass of both the thymus and spleen, and absolute total cell number in both the thymus and spleen (Figure 4a). We also noticed that with the increased proportion of T cells in the spleen (Figure 5a), the proportion of B cells (Figure 6a, top panels) and absolute B cell number (data not shown) in the spleen were significantly decreased. This led us to determine whether defects in B cell development were due to ectopic expression of *FoxN1*^Tg^ mediated by uCreER^T^, which should ubiquitously induce *FoxN1*^Tg^ expression in a wide range of tissues^28^ including hematopoietic cells. We analyzed B cells in the BM, which is the site for B cell development, and found that the proportion of B cells in the BM was indeed decreased (Figure 6a, bottom panels).

In order to confirm the expression of FoxN1 in the BM of *FoxN1*^Tg^uCreER^T+^ mice, we checked FoxN1 mRNA level with real-time RT-PCR. We flushed and collected total BM from femurs, depleted red blood cells, then isolated total mRNA with TRIzol reagent, and digested any transgenic DNA contamination via DNase-I enzymatic treatment. Because transgenic FoxN1 cDNA is characterized by the absence of introns in the genomic DNA sequence, PCR products generated using intron-spanning primers cannot differentiate the transgenic cDNA, which is already incorporated into the genomic DNA of the transgenic mice. Therefore, complete removal of DNA with DNase-I before reverse transcription is critical. We determined that FoxN1 was markedly expressed in the BM of *FoxN1*^Tg^uCreER^T+^ juvenile mice (Figure 6b the rightmost bar). However we did not detect FoxN1 expression in the BM of WT control mice, by comparing the WT spleen, which should not have any FoxN1 expression (Figure 6b). The results indicate that *FoxN1*^Tg^uCreER^T+^ mice have ectopic FoxN1 expression in the BM following the deletion of *STOP*^flox^, mediated by uCreER^T^.

### Overexpression of exogenous *FoxN1*^Tg^, mediated by K5CreER^T^, has different effects in adult and juvenile mice

As K5 is a partner of K14 with the same localization and similar characteristics,^36,37^ we crossbred *Rosa26*-*STOP*^flox^–*FoxN1* mice with K5CreER^T^ mice,^25^ which express K5-deriven Cre-recombinase upon TM induction. We did not observed postnatal *FoxN1* gain-of-function mutation-induced newborn lethal phenotype, and the mice have a normal lifespan.

**Figure 7:**
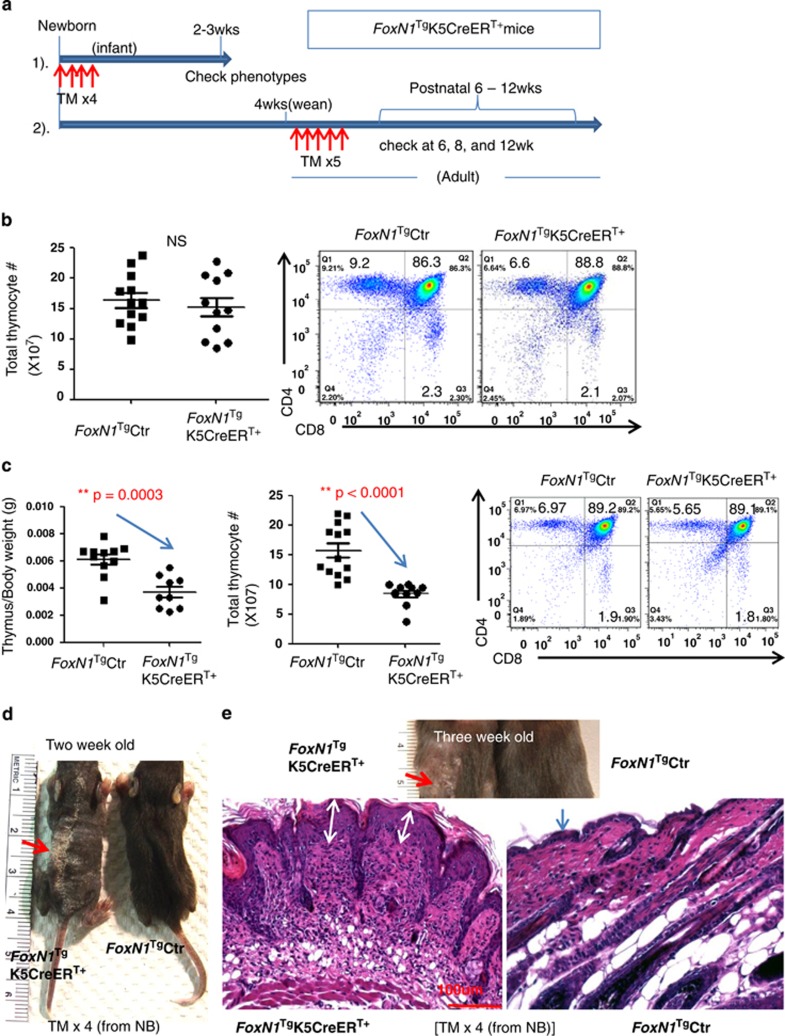
FoxN1 overexpression mediated by K5CreER^T+^ produced different outcomes in newborn and adult thymus and skin. (**a**) Schematic of induced *FoxN1*^Tg^ expression with TM in *FoxN1*^Tg^K5CreER^T+^ newborn (strategy 1) and adult (strategy-2) mice; (**b**) the panel on the left shows summary of absolute total thymocyte numbers from 11 *FoxN1*^Tg^K5CreER^T+^ and 11 *FoxN1*^Tg^K5CreER^T^^−^ control adult mice, injected with TM x5 from adult (see TM induction strategy-2, shown in panel **a**), whereas flow cytometric dot plots on the right show a representative profile of CD4^+^ *versus* CD8^+^ thymocytes; (**c**) the two leftmost panels show decreased thymic weight and total thymocyte numbers; the dot plots on the right show a representative profile of CD4^+^ *versus* CD8^+^ thymocytes in *FoxN1*^Tg^K5CreER^T+^ juvenile mice injected with TM x4 from birth (see TM induction strategy 1, shown in panel **a**); (**d**) skin profile of *FoxN1*^Tg^K5CreER^+^ 2-week-old; and (**e**) 3-week-old juvenile mice, injected with TM x4 from birth (same as panel **c**). Image shows ichthyosis-like, coarse skin and hair loss (red arrow) in a representative *FoxN1*^Tg^K5CreER^T+^ juvenile mouse. Panel **e** bottom shows representative H&E staining of microstructure of the skin from a representative *FoxN1*^Tg^K5CreER^T+^ 3-week-old juvenile mouse. Double headed white arrows indicate increased thickness in the epidermal layer. **=statistically very significant

We found, by observing GFP expression in juvenile (2 weeks) thymuses, that Cre-recombinase leakage in K5CreER^T^ was not as strong as in uCreER^T^ (Supplementary Fig. S3). Therefore, we intraperitoneally injected TM into these mice to enhance induction of *FoxN1*^Tg^ expression in *FoxN1*^Tg^K5CreER^T+^ newborn and adult (4–5 weeks) mice (described in Figure 7a). In adult mice we found that following five-time (5 × ) TM-injections, the *FoxN1*^Tg^ did not result in any adverse phenotypes in the skin and thymus (mass, total thymocyte number, and CD4 *versus* CD8 profile, Figure 7b). However, in juvenile mice, after x4 TM-injections at the neonatal stage, mild adverse phenotypes were observed in the juvenile thymus, such as reduced thymic mass, total thymocytes, and all subset cell numbers (data not shown), but there was no change in CD4 *versus* CD8 profile (Figure 7c). However, this induction strategy induced a severe skin phenotype, exhibiting ichthyosis-like coarse skin and hair loss phenotypes (Figures 7d and e). The results suggest that: (1) *FoxN1*^Tg^ overexpression in the juvenile stage may adversely influence the underdeveloping epithelium, whereas it may not affect or may even be beneficial for mature epithelial tissues in adults;^15^ (2) this adverse effect is more severe in the skin than in the thymus.

## Discussion

The biological significance of inborn and postnatal loss-of-function mutations in the *FoxN1* gene has been studied for several decades.^4,8, 9, 10, 11,38,39^ However, the biological significance of inborn and postnatal *FoxN1* gain-of-function mutations has just begun to be probed.^12,14,15,20,24^ In this report, we used our newly developed inducible tissue-specific mouse model for *FoxN1* gain-of-function mutations to establish biologically significant evidence that over- and ectopic-expression of FoxN1 in early life influences immature TEC, T and B cell, and skin epithelial development. We found that K14Cre-mediated *Rosa26-STOP*^flox^–*FoxN1*^Tg^ newborns had a neonatal lethal phenotype caused by dehydration due to abnormal permeability in the skin and defect in nursing. These neonatal mice shared similar phenotypes with the involucrin promoter-driven *FoxN1* transgenic neonatal mice,^24^ which have enhanced ectopic expression of FoxN1. The uCreER^T^-mediated overexpressed *FoxN1* induced by progressive leakage of Cre-recombinase beyond the neonatal stage affected the development of the thymus, thymocytes, and BM in the juvenile stage, indicating that TEC development skewed towards mTECs, thymocyte numbers were significantly decreased in all subsets, a proportion of peripheral T cells was increased but the absolute number and functional response were significantly reduced, and the numbers of BM and peripheral B cells were significantly decreased. However, K5CreER^T^-mediated *FoxN1* overexpression via TM induction in the adults resulted in normal thymus and skin, whereas mice subjected to TM induction as neonates had mildly abnormal development of the thymus, and severe ichthyosis-like skin in the juvenile mice.

Generally, enhancing FoxN1 expression in an aged thymus should rejuvenate atrophied dysfunction, as FoxN1 is decreased with age.^12,14,15,19^ However, no reports thus far looked into whether enhanced FoxN1 expression in the juvenile thymus, which already has high levels of FoxN1, is beneficial for further thymic development. Our report provides a negative answer, in which *FoxN1* gain-of-function mutations in juvenile mice (younger than 3 weeks, before weaning) had an adverse effect on thymic development. This adverse effect on the skin occurred early (neonatal stage) and was severe (lethality). Interestingly, these lethal phenotypes were not reported in K14 and K5 promoter-driven *FoxN1* transgenic mice made by other groups.^14,40^ This is probably because we used both the *Rosa26* promoter to ensure the *FoxN1* gene is expressed in all Rosa26 positive tissues (almost ubiquitously), followed by pCAG promoter^21, 22, 23^ to ensure strong FoxN1 expression. The pCAG promoter is a CMV-IE enhancer plus chicken *β*-actin promoter sequence,^21^ which was confirmed to be able to drive enhanced gene expression,^41^ particularly in the cutaneous epithelium,^21^ which may explain why the skin phenotype is so strong. However, upregulating FoxN1 in later life stage via inducible p*CAG*-*FoxN1ER* mediated by *FoxN1*-Cre Tg, which achieves overexpressing exogenous FoxN1 only in endogenous FoxN1-declined TECs, is beneficial to rejuvenation of aged thymic function.^15^

We did not observe defects during thymic development induced by *FoxN1* gain-of-function mutations in the prenatal thymus in our K14-, K5-, and ubiquitous Cre-mediated FoxN1 overexpressing mice. Therefore, the dosage of FoxN1 could be considered ‘the more, the better' in the prenatal thymus. However, *FoxN1* gain-of-function mutations in the early postnatal thymus resulted in defects in TEC development, as observed in the ubiquitous CreER^T^ (continuous Cre-recombinase leaky)-mediated and K5CreER^T^ (juvenile mice with TM x4)-mediated *Rosa26-STOP*^flox^–*FoxN1*^Tg^ mice (Figures 2–4 and 7c). Therefore, the dosage of FoxN1 could not be considered ‘the more, the better' for juvenile stage thymic development. At this stage, the thymic medulla is still undergoing development,^30^ and expression of FoxN1 may be just beginning to decline in the natural thymus, where enhancing FoxN1 expression may break this balance. However, in the adult and late postnatal thymus, FoxN1^+^ TECs are markedly reduced.^42,43^ Enhancing FoxN1 expression may produce a beneficial effect that may slow down thymic aging,^14,15^ or at least no harmful effects as shown in our K5CreER^T^ (with TM x5 induction in the adult)-mediated *Rosa26-STOP*^flox^–*FoxN1*^Tg^ transgenic adult mice (Figure 7b).

In our uCreER^T^-mediated *Rosa26-STOP*^flox^–*FoxN1*^Tg^ transgenic juvenile mice, we also found that B lymphopoiesis was markedly decreased in the BM and spleen (Figure 6a). This is consistent with the finding in K14 promoter-driven *FoxN1* transgenic mice, in which B-lineage cell numbers were significantly lower.^20^ It could be caused by exogenous FoxN1 expression in nonprominent locations, not only in BM mesenchymal origin stromal cells but also in hematopoietic origin cells because of ubiquitous CreER^T^.

In our model, FoxN1 overexpression in the BM not only disrupted B lymphopoiesis, but was also not beneficial for T cell development. In the uCreER^T^ leakage-mediated FoxN1 overexpression in *Rosa26-STOP*^flox^–*FoxN1*^Tg^ juvenile mice, BM LSK (lineage negative, Sca-1^+^ and c-kit^+^) cells, which are T-lymphohematopoietic progenitors in the BM, were decreased (Supplementary Figure S4) and early T-cell thymic progenitor (lineage negative, CD44^+^CD25^−^, and c-kit^+^) cells were increased in percentage (data not show), but decreased in absolute number along with all other thymocyte subpopulations (Figure 4). At least, FoxN1 expression in BM stromal (niche) cells is not critically required for T-cell development, as WT thymic lobes grafted into nude (*FoxN1*^null^) mice under the kidney capsule, without any modification in the *FoxN1*^null^ host BM niche, are able to generate normal T cells, which is a common model for studying lymphostromal interactions.

The *FOXN1* leads to congenital alopecia and an alymphoid thymus with severe combined primary T-cell immunodeficiency,^7,44, 45, 46^ resulting in death in early childhood from severe infections.^44,47^ Therefore, gene therapy with *FOXN1* is one of options to be possibly selected to treat fetal *FOXN1* mutations. If so, the dosage of *FOXN1*, target tissues, and developmental stages must be carefully considered, because our data has demonstrated that over- and ectopic-expression of FoxN1 is pathogenic and potentially lethal. In addition, although there are not any reported diseases predicated on the over- and ectopic-expression of FoxN1, this does not rule out their discovery in the near future. A growing paradigm of microRNAs regulating gene activity is emerging. A recent report has linked silencing of microRNAs (miR-18b and miR-518b) with the upregulation of FoxN1 in embryonic stem cells.^48^ Evidence to support microRNA mutations affecting gene expression and leading to heritable diseases is emerging with time. Our results provide a clue of potential pathologies that could emerge from mutations in the microRNA or microRNA target regulators that maintain FoxN1 levels.

It is taken for granted that loss-of-function mutations induce defective phenotypes, whereas it is hard to accept that gain-of-function mutations can also induce defective phenotypes. Our results show that neonatal overexpression of FoxN1 mainly affects skin development, which was also demonstrated by an *Involucrin* promoter-driven *FoxN1* transgenic mouse model.^24^ Our study addressed whether FoxnN1 expression in the thymus can be considered ‘the more, the better'. Although it is not the case in certain developmental stages, such as in juveniles, enhancing FoxN1 expression in the FoxN1-declined aged thymus should provide significant rejuvenation.^12,14,15^ In fact, ectopic-expression of FoxN1 in tissues that do not predominantly express FoxN1, such as the BM, is harmful. The biological significance of gain-of-function mutations in the *FoxN1* gene is summarized in Supplementary Figure S5. Ultimately, if FoxN1 is not expressed in the right tissues and right life developmental stages, then physiological abnormalities will be induced. In our models, FoxN1 gain-of-function mutations disrupted B-lymphopoiesis and did not help T lymphopoiesis. FoxN1 possesses developmental stage and tissue-specific activity.

## Materials and Methods

### Mice, age groups, genotyping, and animal care

Neonatal (1 day) and juvenile (2–3 weeks) mice (C57BL/6 genetic background) were used. *Rosa26-STOP*^flox^–*FoxN1* transgenic (termed *FoxN1*^Tg^) mice were generated by our laboratory (available upon request),^1^ and genotyped with three primers (Supplementary Figure S1A). The *FoxN1*^Tg^ mice were crossbred with K14Cre,^49^ K5CreER^T^^25^ and ubiquitous pCAG-CreER^T^ (uCreER^T^)^28^ mice (Jackson Laboratories, Bar Harbor, ME, USA, #004782, #018394, and #004682). The *FoxN1*^flox^ (fx) mice carrying a TM-inducible uCreER^T^ (termed fx/fx-uCreER^T^) were generated and genotyped as described previously^11^ (Jackson Laboratories #012941). All animal experiments were performed according to the protocols approved by the Institutional Animal Care and Use Committee of the University of North Texas Health Science Center, in accordance with guidelines from the National Institutes of Health, USA.

### Skin permeability assay

Details are described in a previous publication.^26^ In brief, freshly isolated embryos were rinsed in phosphate buffered saline (PBS) and immersed in X-gal staining reagent overnight at 30 °C. On the following day, embryos were rinsed in PBS and photographed.

### IF staining

Cryosections (5/6 *μ*m thick) were stained as described previously.^50^ The primary antibodies used were rabbit anti-FoxN1 (provided by Dr. Itoi),^43^ anti-claudin-3,4 (Invitrogen, Grand Island, NY, USA, #34-1700 and #36-4800), anti-Keratin-5 (Covance, Princeton, NJ, USA, #PRB-160P), Biotinylated-UEA-1 (Vector Laboratories, Burlingame, CA, USA, #B-1065), anti-Aire (Santa Cruz, Dallas, TX, USA, #SC-33189, anti-*β*5t (Medical and Biological Laboratories, Nagoya, Japan, #PD021), and anti-Keratin-8 (Troma-1 supernatant). The secondary antibodies used were Cy3-conjugated or Alexa-Fluor-488-conjugated donkey anti-rabbit or -rat IgG (Jackson ImmunoResearch Laboratories, West Grove, PA, USA).

### Real-time RT-PCR

Total RNA from mouse thymus, spleen, and BM was isolated with TRIzol reagent and reverse transcribed to cDNA with the SuperScriptIII cDNA kit (Invitrogen). Real-time RT-PCR was performed with TaqMan reagents. The sequences of FoxN1 primers and probe (TaqMan method) were as described previously.^11^ The relative expression levels of FoxN1 mRNAs from the thymus, spleen, and BM were internally normalized to GAPDH levels, then compared with a *ΔΔ*C_T_ value from pooled young WT thymuses. This *ΔΔ*C_T_ value was always arbitrarily set as 1.0 in each real-time PCR reaction.

### Western blot analysis

The whole thymus was subjected to homogenization and protein extraction in RIPA lysis buffer (Sigma, St. Louis, MO, USA, #R0278). Protein, ~25 *μ*g/lane, was loaded under reducing conditions for direct Western blot assay with FoxN1^43^ (81 KD band) and GFP (monoclonal, Santa Cruz, #SC-9996, 27 KD band) antibodies. GAPDH or *β*-actin were used as an internal loading control.

### Flow cytometry assays

Single cell suspensions of thymocytes, spleen, and BM (flushed from the mouse femur) cells, as well as TECs (isolated with collagenase-V/DNase-I isolation method)^50^ were stained with combinations of fluorochrome-conjugated antibodies against cell surface markers and/or an intracellular marker, which are indicated in each figure. Data was acquired using a BD LSRII Flow Cytometer (BD Bioscience, San Jose, CA, USA) and analyzed using FlowJo software (Tree Star, Inc., Ashland, OR, USA).

### Analysis of intracellular IL-2 in peripheral CD4^+^ T cells in response to costimulation of CD3 and CD28 antibodies

Red blood cell lysis buffer (Sigma, #R7757) treated spleen cells (2 × 10^6^ per well) were cultured with anti-mouse CD3*ɛ* and CD28 antibodies (2 *μ*g/ml each) supplemented with BD GolgiStop (0.7 *μ*l/ml, from BD Biosciences, #554724) for 5 h. The harvested cells were stained for CD4 on the surface, fixed and permeabilized,^12^ then stained with fluorochrome-conjugated IL-2 antibody intracellularly, following flow cytometry assay.

### Statistics

Statistical significance was analyzed by unpaired Student's *t*-test. Differences were considered statistically significant at values of *P*<0.05.

## Abbreviations

BM: bone marrow
cTEC/mTEC: cortical/medullary thymic epithelial cells
fx: *loxP*-floxed-*FoxN1*
K14 or K5: keratin-14 or keratin-5
uCreER^T^: ubiquitous promoter-driven Cre-recombinase and estrogen-receptor fusion protein
WT: wild type
