# Supplementary material for: Biological significance of FoxN1 gain-of-function mutations during T and B lymphopoiesis in juvenile mice
Source: Cell Death Dis. 2014 Oct 9;5(10):e1457–. doi: 10.1038/cddis.2014.432 (PMC4237256; doi:10.1038/cddis.2014.432)
Supplement: Supplementary Figures [file cddis2014432x1.pdf]

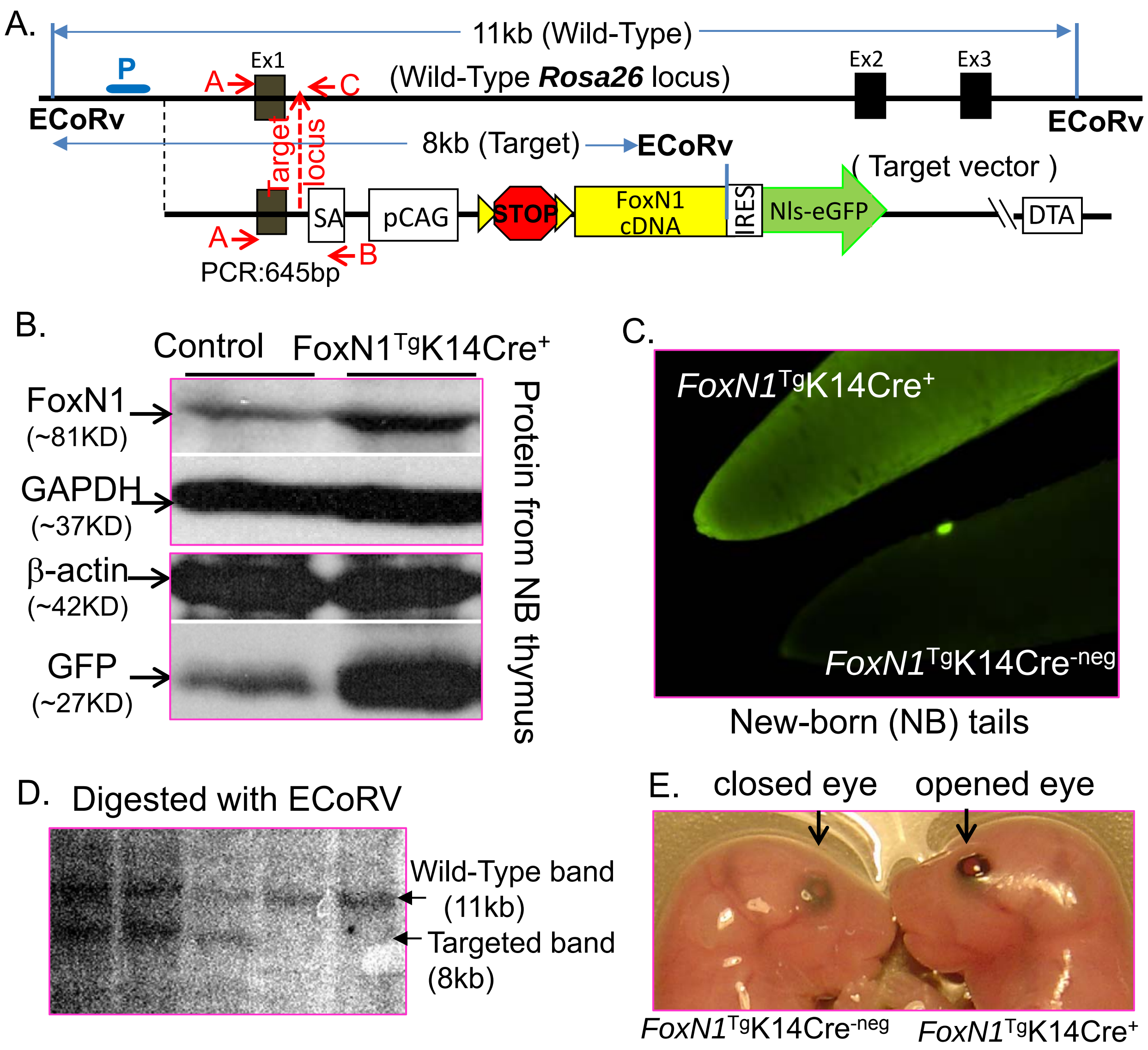

Supplemental Figure S1. The generation of *Rosa26-STOP*^{flox}*-FoxN1*^{Tg} mice and confirmation of correct targeting. (A) Schematic diagram of *Rosa26-STOP*^{flox}*-FoxN1*^{Tg} gene targeting and elements of DNA insertion, as well as locations of genotyping PCR primers (red arrows). The genotyping primers are: forward Primer A on *Roas26* gene promoter: 5'-ggc cct tgc cat tgg ctc gt-3'; reverse Primer B on the target insertion: 5'-tcc cca ctg gaa aga ccg cga-3'; and reverse Primer C on *Rosa26* gene just behind the target locus: 5'-gcc ctc gct cct tcc ctc ca-3'. These primers produce amplicons: 645-bp representing primers A-B product, and 293-bp wild type (WT) representing primers A-C product, respectively. **(B)** A representative Western blot result shows increased FoxN1 and GFP expression in *Rosa26-STOP*^{flox}*FoxN1*^{Tg}*-K14Cre*$^{+}$ and *-K14Cre*$^{-neg}$ control mice. The experiment was performed at least 5 times with more than 5 mice/group, producing consistent results. **(C)** Green fluorescence is detected in *Rosa26-STOP*^{flox}*FoxN1*^{Tg}*-K14Cre*$^{+}$ (top), but not in control *Rosa26-STOP*^{flox}*FoxN1*^{Tg}*-K14Cre*$^{-neg}$ neonatal tail (bottom). **(D)** A representative Southern blot result shows WT EcoRV digested band (11Kb) and gene targeted band (8Kb), confirming correct gene targets at *Rosa26* gene locus. **(E)** Opened eyes (right) can be observed in *Rosa26-STOP*^{flox}*FoxN1*^{Tg}*-K14Cre*$^{+}$, but not in control *Rosa26-STOP*^{flox}*FoxN1*^{Tg}*-K14Cre*$^{-neg}$ (left) gestation E18-19 mice.

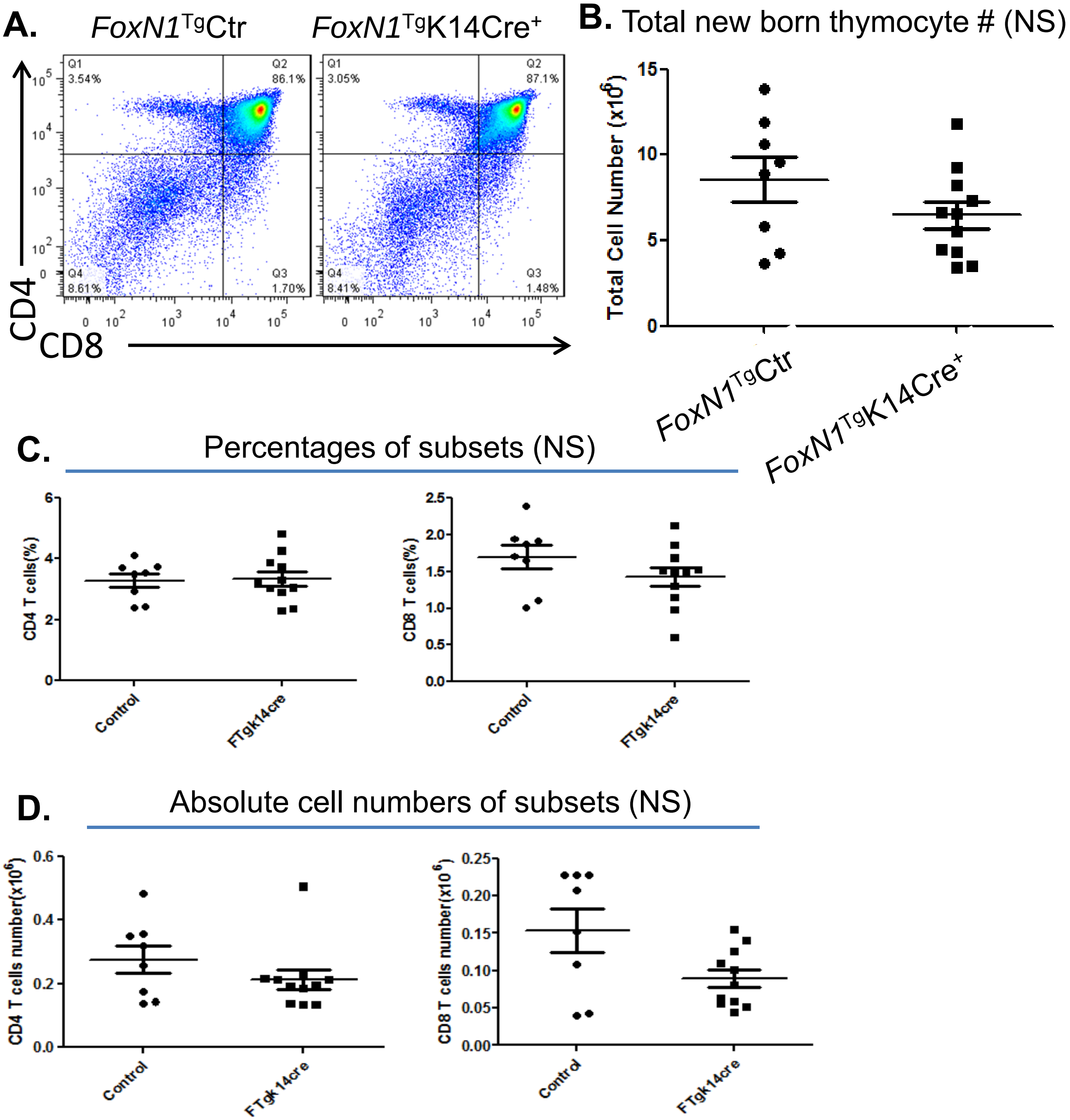

Supplemental Fig.S2. Flow cytometric results of thymoyctes from *Rosa26-STOP*^{flox}*FoxN1*^{Tg}*-K14Cre*$^{+}$ and *-K14Cre*$^{-neg}$ newborn mice, showing no difference in CD4 versus CD8 profile **(A)**, total thymocyte numbers **(B)**, percentages of CD4 single positive (SP) or CD8 SP thymocytes **(C)**, and absolute cell numbers of CD4SP and CD8SP **(D)** between these two groups.

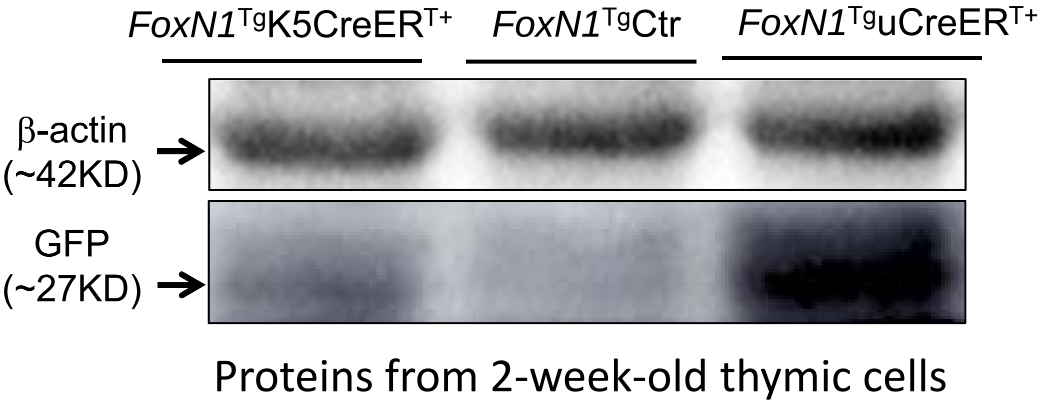

Supplemental Fig. S3. Comparison of uCreER^{T} and K5CreER^{T} leakage in the thymuses of *FoxN1*^{Tg}uCreER^{T} and *FoxN1*^{Tg}K5CreER^{T} 2-week-old juvenile mice.

A representative western blot result shows expression of GFP in the thymuses of *FoxN1*^{Tg}uCreER^{T} and *FoxN1*^{Tg}K5CreER^{T} mice, two weeks after birth without tamoxifen induction. Density of the GFP bands represents levels of CreER^{T} leakage, induced by removal of roadblock (*STOP*^{flox}). *FoxN1*^{Tg}Ctr mice (carry *STOP*^{flox}*FoxN1*^{Tg} cassette, but do not contain any Cre-recombinase -- neither uCreER^{T} nor K5CreER^{T}) are for background control. This experiment was repeated at least three times with three animals in each group producing consistent results.

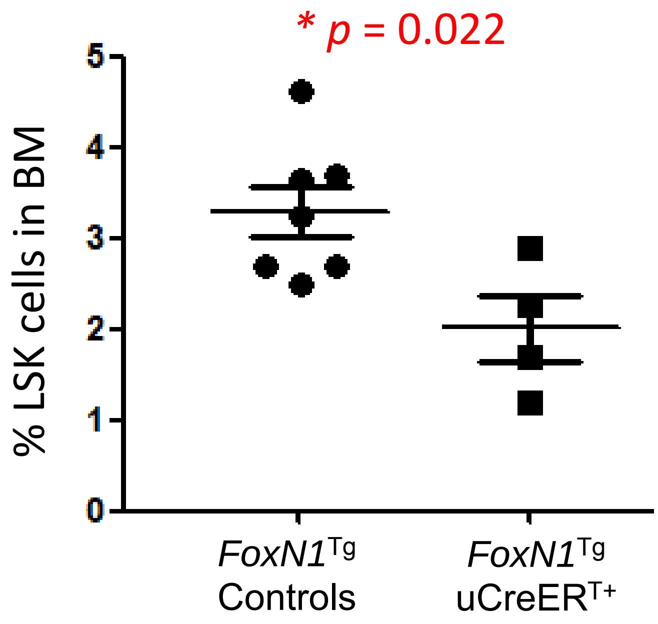

Supplemental Fig. S4. Bone marrow (BM) LSK cells were decreased in *FoxN1*^{Tg}uCreER^{T+} BM of juvenile mice.

The result shows that percentage of LSK (Lin^{-neg}, Sca1^{+}cKit^{+}) cells, which are T-lymphohematopoietic progenitors in the BM, were significantly decreased in *FoxN1*^{Tg}uCreER^{T+} compared to *FoxN1*^{Tg} control (uCreER^{T} negative) juvenile (20 days after birth) mice. Each spot represents one animal.

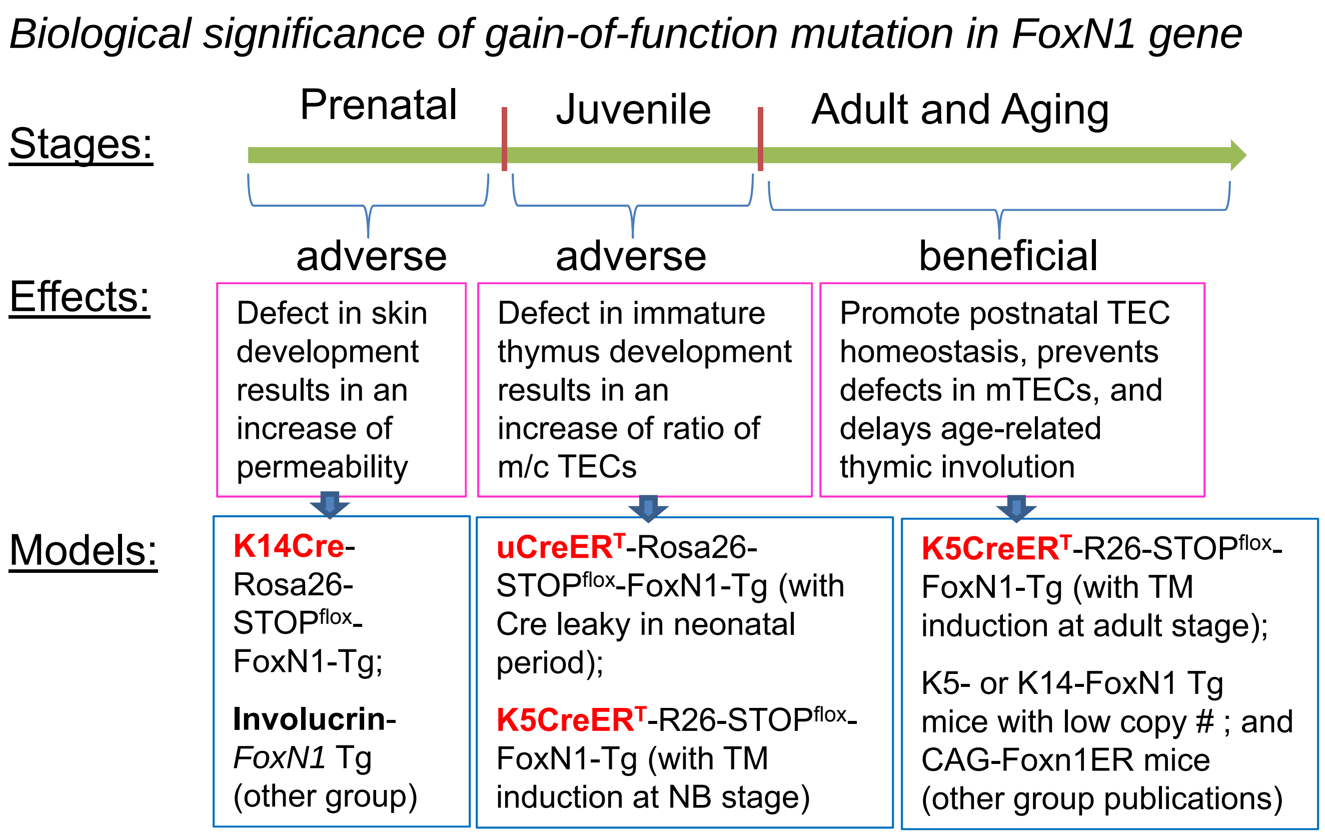

Supplemental Fig. S5. Summary of biological significance of gain-of-function mutations in the *FoxN1* gene with various genetically engineered mouse models.

Gain-of-function mutations in *FoxN1* usually result in adverse phenotypes in early life stages. Generally, *FoxN1* gain-of-function mutations in the prenatal stage induced defects in skin development, exhibiting ichthyosis-like skin, but did not induce defects in thymus development. However, *FoxN1* gain-of-function mutations during the juvenile stage induced defects both in skin and thymus development. When the mice enter adulthood, it seems that FoxN1 over-expression does not affect the skin or the thymus. On the other hand, it may be beneficial in attenuating thymic aging.
